# Gene Expression Correlation for Cancer Diagnosis: A Pilot Study

**DOI:** 10.1155/2014/253804

**Published:** 2014-04-09

**Authors:** Binbing Ling, Lifeng Chen, Qiang Liu, Jian Yang

**Affiliations:** ^1^Drug Discovery and Development Research Group, College of Pharmacy and Nutrition, University of Saskatchewan, 107 Wiggins Road, Saskatoon, SK, Canada S7N 5E5; ^2^Vaccine and Infectious Disease Organization-International Vaccine Centre, University of Saskatchewan, 120 Veterinary Road, Saskatoon, SK, Canada S7N 5E3

## Abstract

Poor prognosis for late-stage, high-grade, and recurrent cancers has been motivating cancer researchers to search for more efficient biomarkers to identify the onset of cancer. Recent advances in constructing and dynamically analyzing biomolecular networks for different types of cancer have provided a promising novel strategy to detect tumorigenesis and metastasis. The observation of different biomolecular networks associated with normal and cancerous states led us to hypothesize that correlations for gene expressions could serve as valid indicators of early cancer development. In this pilot study, we tested our hypothesis by examining whether the mRNA expressions of three randomly selected cancer-related genes *PIK3C3*, *PIM3*, and *PTEN* were correlated during cancer progression and the correlation coefficients could be used for cancer diagnosis. Strong correlations (0.68 ≤ *r* ≤ 1.0) were observed between *PIK3C3* and *PIM3* in breast cancer, between *PIK3C3* and *PTEN* in breast and ovary cancers, and between *PIM3* and *PTEN* in breast, kidney, liver, and thyroid cancers during disease progression, implicating that the correlations for cancer network gene expressions could serve as a supplement to current clinical biomarkers, such as cancer antigens, for early cancer diagnosis.

## 1. Introduction

Cancer is a malignant neoplasm that kills about 75,500 Canadians in 2013 [1]. Taking advantage of recent progress in cancer treatments, especially in chemotherapy and targeted therapy, the age-standardized five-year relative survival rate (RSR) for Canadian cancer patients has increased from 56% in the 1992–1994 period to 63% in the 2006–2008 period [1]. However, prognosis for high-grade, late-stage, and recurrent cancers remains poor; for example, the five-year RSR for breast cancer dropped dramatically from 96% for stage I to 26% for stage IV [2]. Therefore, early diagnosis is still essential for cancer patient survival.

Cancer is characterized by uncontrolled cell growth as a consequence of activating protooncogenes and/or inactivating tumor suppressor genes [3–5]. Searching for consistently up- or downregulated genes, proteins, or clusters of genes has been the mainstream in identifying potential biomarkers for early cancer diagnosis [6–8]. Nevertheless, this approach experiences a key limitation that expression of cancer-related genes and proteins may change dramatically during disease progression, which could lead to misdiagnosis. Cancer cells are living in a hostile environment and undertaking constant attacks by the human immune system [9, 10]. They need to network and coordinate many genes to counteract the host attacks for their own survival, and one of such networking examples is the crosstalk between different signaling pathways to enhance cancer proliferation, migration, and drug resistance [11–15]. With recent advances in high-throughput genomic and proteomic studies and significantly increased computing power, constructing and dynamically analyzing biomolecular networks to detect tumorigenesis, predict cancer progression, and even possibly guide cancer treatment has attracted a significant amount of research interest in recent years [16–19].

Tumorigenesis as well as cancer progression is a complex and dynamic process. A recent study on protein interaction networks showed that the biomolecular networks for cancer cells may be significantly different from those for normal cells [20]. This observation led us to hypothesize that increased correlations for the expressions of genes in the cancer networks associated with decreased correlations for the expressions of genes in the normal networks might serve as valid biomarkers for early diagnosis of tumorigenesis and cancer progression. In this pilot study, we randomly chose three cancer-related genes,* PIK3C3*,* PIM3*, and* PTEN*, to test our hypothesis without knowing whether they are clustered together in any cancer network prior to a costly large scale patient-population based and cancer-network confirmed clinical screening.* PIK3C3* encodes class III phosphatidylinositol 3-kinase (PIK3C3/Vps34), which works together with SH3GLB1/Bif-1, a haploinsufficient tumor suppressor, to form autophagosome to counteract oncogene-driven tumorigenesis [21, 22].* PIM3* is an oncogene encoding a Ca^2+^/calmodulin-dependent protein kinase Pim-3, which inhibits cell apoptosis and promotes cell survival [23–25].* PTEN* is a tumor suppressor gene that encodes PTEN (phosphatase and tensin homologue deleted from chromosome-10). PTEN catalyzes the dephosphorylation of PI(3,4,5)P3 (phosphatidylinositol 3,4,5-trisphosphate) to PI(3,4)P2 (phosphatidylinositol 3,4-bisphosphate), which, in turn, downregulates the AKT pathway to inhibit cell growth and induce cell apoptosis [26–28]. Although the three selected genes are neither clustered directly in any identified signaling pathway nor confirmed to be copresent in any cancer network, our limited but insightful analysis showed high correlations for the mRNA expressions among the three genes in breast cancer, between* PIK3C3* and* PTEN* in ovary cancer, and between* PIM3* and* PTEN* in kidney, liver, and thyroid cancers during disease progression.

## 2. Materials and Methods

The 96-sample TissueScan Oncology qPCR Cancer Survey Panel was purchased from the OriGene Technologies, Inc. (Rockville, MD, USA). Primer sequences for* PIK3C3*,* PIM3*, and* PTEN* (Table 1) were designed and synthesized by Applied Biosystems (Foster City, CA, USA) based on the ITS region. The TaqMan probe was labeled with FAM at 5′-end and with nonfluorescent quencher at 3′-end. The mRNA expression levels for genes* PIK3C3*,* PIM3*, and* PTEN* were evaluated with the 96-sample qPCR Cancer Survey Panel by quantitative real-time RT-PCR using an Applied Biosystems 7300 Real-Time PCR System. The expression for each gene was normalized to the internal control, *β*-actin, in different patients. The quantitative real-time RT-PCR reaction mixture consisted of TaqMan Gene Expression Master Mix (Applied Biosystems), 0.9 *μ*M of each primer for gene* PIK3C3*,* PIM3,* or* PTEN* and the* ACTB* gene encoding *β*-actin, and 0.9 *μ*M of the TaqMan probe. The PCR reaction mixture was then added to the 96-sample qPCR Cancer Survey Panel at 30 *μ*L per well. The amplification was carried out under the following conditions: 2 min at 50°C, 10 min at 95°C, 60 cycles of 15 s at 95°C, and finally 1 min at 60°C. The expression level of each gene was averaged by disease stage and normalized to *β*-actin. The fold difference in mRNA expression at each disease stage was determined by comparison to expression levels in patients with noninvasive cancer (stage 0, expression level set as 1). For each cancer type, unpaired *t*-test with Welch's correction between the averaged expression levels in noninvasive and invasive cancer patients was performed with *α* = 0.05 using GraphPad Prism 4.0 (GraphPad Software, San Diego, CA, USA).

## 3. Results and Discussion

Tumor development, progression, metastasis, and drug resistance require a delicately controlled gene network [29–32]. These genes are unlikely to play equally important roles in the network. Constructing and analyzing biomolecular networks using bioinformatic techniques has emerged to be a promising tool in identifying key players in the cancer networks. However, clinical validation of a cancer network is facing several challenges: (1) a large patient population taking into consideration the factors such as race, gender, age, and environment, nutrition, (2) high cost associated with the clinical screening, and (3) heterogeneity of the tumor tissues. Thus, a pilot study is always necessary and beneficial for such type of research.

Recently, Islam et al. reported that the biomolecular networks were significantly different between normal and cancerous states from their protein interaction studies [20]. Based on this report, we hypothesized that increased correlations for the expressions of a set of genes (cancer network) associated with decreased correlations for the expressions of another set of genes (normal network) might serve as an indicator for tumorigenesis and cancer progression. We undertook the current pilot study to test our hypothesis by evaluating the mRNA expressions of three randomly selected cancer-related genes,* PIK3C3*,* PIM3*, and* PTEN*, using quantitative real-time RT-PCR approach. The three selected genes are not directly clustered in any identified signal transduction pathway to our knowledge, and the reason for choosing quantitative real-time RT-PCR is that it is faster, more reliable, more quantifiable, and cheaper than protein analysis of patient samples. Although it is unknown whether the three genes are grouped together in any cancer network, observation of strong correlations among their mRNA expressions would definitely support our hypothesis that gene expression correlations were equally valid as the genes themselves to serve as biomarkers for diagnosis of tumorigenesis and cancer progression.

Ninety-six patient samples were included in this pilot study with twelve samples spreading over different disease stages for each of the eight types of cancer (see Supplementary Table S1 available online at http://dx.doi.org/10.1155/2014/253804). The mRNA expression levels of genes* PIK3C3*,* PIM3*, and* PTEN* were quantitatively analyzed by real-time RT-PCR. As shown in Table 2, the disease stage-averaged mRNA expressions (stages I to IV) for* PIK3C3*,* PIM3*, and* PTEN* were elevated by 3.1-fold (*P* = 0.01), 3.8-fold (*P* = 0.00), and 3.1-fold (*P* = 0.04), respectively, in breast cancer. The disease stage-averaged mRNA expression for* PIK3C3* was downregulated by 2-fold (*P* = 0.03) in thyroid cancer. No statistically significant up- or downregulation of the disease stage-averaged mRNA expressions for the genes was observed in colon, kidney, liver, lung, ovary, and prostate cancers. Neither of the disease stage-averaged mRNA expressions for* PIM3* and* PTEN* was statistically significantly altered in thyroid cancer. These data suggested that disease stage-averaged mRNA expressions for genes* PIK3C3*,* PIM3*, and* PTEN* could be used for breast cancer diagnosis but not for the other types of cancer. Since previous studies have shown upregulation of PIM3 or downregulation of PTEN in various types of cancer [33–36], the disease stage-averaged method may have neglected important information about the mRNA expressions of the three genes.

**Figure 1:**
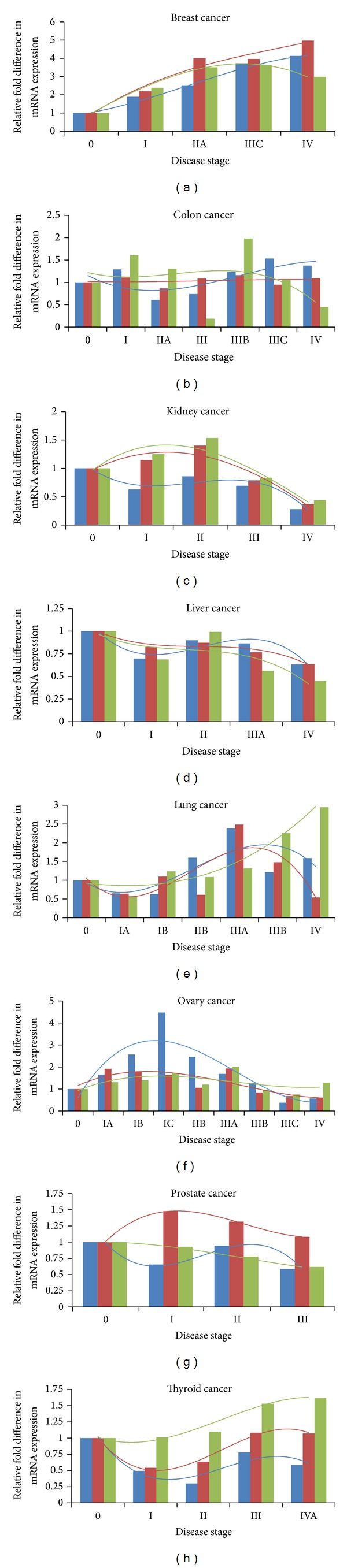
Relative fold difference in mRNA expressions of* PIK3C3* (blue),* PIM3* (coral), and* PTEN* (green) at different disease stages of breast (a), colon (b), kidney (c), liver (d), lung (e), ovary (f), prostate (g), and thyroid (h) cancers. The mRNA expression for each gene was trended using a third-degree polynomial function over the disease progression. Fold difference in mRNA expression at each disease stage was determined by comparison to the expression level in noninvasive cancer patients (stage 0, expression level set as 1).

In order to get a better understanding on the expressions of* PIK3C3*,* PIM3*, and* PTEN*, we compared their mRNA expression levels at each disease stage (Figure 1). The mRNA expressions of all three genes varied significantly over the disease progression. In breast cancer, the mRNA expression levels of* PIK3C3* and* PIM3* were steadily increased during disease progression and elevated by 4.1- and 5.0-fold, respectively, at stage IV. The mRNA expression level of* PTEN* was also augmented at each disease stage with a maximum of 3.6-fold increase at stage IIIC. In colon cancer, the mRNA expression of* PIK3C3* decreased by about 40% at stage IIA and then gradually increased with a maximum of 50% upregulation at stage IIIC. The mRNA expression of* PIM3* was not altered over the disease progression; however, the mRNA expression of* PTEN* fluctuated with a significant 5-fold decrease at stage III. In kidney cancer, the mRNA expression of* PIK3C3* slowly decreased as the disease progressed, whereas the mRNA expressions of* PIM3* and* PTEN* were both bell-shaped with a maximum of 40% and 50% increases, respectively, at stage II. As the disease approached stage IV, the mRNA expression levels of all three genes were downregulated by at least 2-fold. In liver cancer, the change of the mRNA expression levels of the three genes was not significant except for stage IV. At stage IV, the expression level of* PTEN* was decreased by 2-fold. In lung cancer, both* PIK3C3* and* PIM3* gave a bell-shaped response towards disease progression with a maximum of 2.4- and 2.5-fold increases, respectively, at stage IIIA, whereas the mRNA expression of* PTEN* increased continuously through the disease progression and reached a 2.9-fold elevation by stage IV. In ovary cancer, the expression of* PIK3C3* also exhibited a bell-shaped response towards disease progression with a 4.5-fold increase in stage IC and a 2-fold decrease in stage IV. In prostate cancer, the mRNA expression levels of* PIK3C3* and* PTEN* were slightly downregulated and the mRNA expression level of* PIM3* was slightly upregulated for stages I to III. In thyroid cancer, the mRNA expressions of* PIK3C3* and* PIM3* decreased dramatically in stages I-II and subsequently increased as the disease progressed. The mRNA expression level of* PTEN* was elevated through all disease stages with a maximum of 60% increase at stage IV. From the above analysis, a better understanding about the involvement of genes* PIK3C3*,* PIM3,* and* PTEN* in cancer development and progression was achieved by comparing their mRNA expressions at each disease stage of different types of cancer. It should be noteworthy that mRNA upregulation of tumor suppressor gene* PTEN* warrants neither increased protein level for PTEN as increases in mRNA expression do not necessarily translate proportionally into protein expression nor PTEN in the wild-type as silencing of tumor suppressor genes through mutations is very common in cancers. Although we could determine PTEN expression at the protein level by Western blot and the identity of the PTEN protein (wild-type or mutant protein) by protein sequencing in our research laboratory, it would be costly, time-consuming, and even unnecessary to do that for each cancer patient during clinical laboratory tests when mRNA changes were employed for cancer diagnosis.

Finally, we examined whether the mRNA expressions of* PIK3C3*,* PIM3,* and* PTEN *were correlated during cancer progression. The mRNA expression levels of these three genes were trended using a third-degree polynomial function (Figure 1), and their pair-wise correlation coefficients for both stages 0–IV and stages 0–III were calculated (Table 3). The small sample size associated with each cancer type precluded a complete analysis; nonetheless, valuable information was obtained from our limited analysis. Based on disease stages 0–IV and following the correlation classification defined by Taylor [37], high correlations (0.68 ≤ *r* ≤ 1.0) were observed between* PIK3C3* and* PIM3* in breast cancer, between* PIK3C3* and* PTEN* in breast and ovary cancers, and between* PIM3* and* PTEN* in breast, kidney, liver, and thyroid cancers. Moderate correlation (0.34 ≤ *r* ≤ 0.67) was detected between* PIK3C3* and* PIM3* in ovary cancer. Since RSR is much lower in patients with metastatic cancer, we compared the correlation coefficients for the mRNA expressions of the three genes between stages 0–IV and stages 0–III (Table 3) in an attempt to find out whether these correlation coefficients could provide any hint of cancer metastasis. The change from statistically insignificant moderate correlation for stages 0–III to statistically significant high correlation for stages 0–IV for the mRNA expressions of* PIM3* and* PTEN* was observed in kidney and thyroid cancers, suggesting that the change in the correlation coefficient for the mRNA expressions of* PIM3* and* PTEN* might be able to predict metastasis in kidney and thyroid cancers.

In summary, by using genes* PIK3C3*,* PIM3,* and* PTEN* as test samples, we showed that constructing biomolecular networks for normal and cancerous states and monitoring the correlations for the expressions of genes in the networks is a promising novel strategy in detecting tumorigenesis, cancer progression, and even cancer metastasis. Further studies involving confirmed biomolecular networks and a larger patient population certainly warrant investigations given the potential benefit for early cancer diagnosis and cancer patient survival. It is also important to address whether the biomolecular networks change during cancer progression in the future studies.

## Supplementary Material

Patient demographic characteristics and cancer stage for the Origene TissueScan Oncology qPCR Cancer Survey Panel 96.

## Figures and Tables

**Table 1:** Primer and TaqMan probe sequences for genes *PIK3C3*, *PIM3,* and *PTEN*.

Gene name	Forward primer sequence	Reverse primer sequence	TaqMan probe sequence
*PIK3C3 *	GCCGATGATGAGGATTTGTTGATG	CCTTCTTGGTAGGTTCCAATCCATT	FAM-CCAGGCTCTCAAATAT
*PIM3 *	CGCTACCACCGCTACCA	CCCACACACCATATCGTAGAGAAG	FAM-ACGCCCAGCGACCAC
*PTEN *	GCACTGTTGTTTCACAAGATGATGT	ACCACAAACTGAGGATTGCAAGT	FAM-CCGCCACTGAACATT

**Table 2:** Average fold difference (FD) in the mRNA expression levels of *PIK3C3*, *PIM3,* and *PTEN* in patients with invasive cancer (stages I–IV) relative to patients with noninvasive cancer (stage 0).

Cancer type	*PIK3C3 *		*PIM3 *		*PTEN *	
	FD	*P* value	FD	*P* value	FD	*P* value
Breast	3.1	0.01	3.8	0.00	3.1	0.04
Colon	1.1	0.98	1.0	0.94	1.1	0.91
Kidney	0.6	0.07	0.9	0.41	1.0	0.52
Liver	0.8	0.38	0.8	0.21	0.7	0.09
Lung	1.3	0.77	1.1	0.83	1.6	0.19
Ovary	1.9	0.62	1.3	0.55	1.3	0.39
Prostate	0.7	0.35	1.3	0.61	0.8	0.21
Thyroid	0.5	0.03	0.8	0.26	1.3	0.23

**Table 3:** Pair-wise comparisons of the mRNA expression levels of *PIK3C3*, *PIM3,* and *PTEN *during disease progression in eight cancer types with high (0.68 ≤ *r* ≤ 1.00), moderate (0.36 ≤ *r* ≤ 0.67), and low (*r* ≤ 0.35) correlations were shown as (∗), (∗∗), and (∗∗∗), respectively. Prostate cancer does not contain stage IV samples.

Cancer type	Gene pair	Correlation coefficient (*r*)	
		Stages 0–IV	Stages 0–III
Breast	*PIK3C3-PIM3 *	0.77 (*P* < 0.05)*	0.76 (*P* < 0.05)*
	*PIK3C3-PTEN *	0.70 (*P* < 0.05)*	0.77 (*P* < 0.05)*
	*PIM3-PTEN *	0.77 (*P* < 0.05)*	0.79 (*P* < 0.05)*

Colon	*PIK3C3-PIM3 *	−0.17 (*P* > 0.05)***	−0.18 (*P* > 0.05)***
	*PIK3C3-PTEN *	0.35 (*P* > 0.05)***	0.41 (*P* > 0.05)**
	*PIM3-PTEN *	−0.22 (*P* > 0.05)***	−0.20 (*P* > 0.05)***

Kidney	*PIK3C3-PIM3 *	0.52 (*P* > 0.05)**	−0.30 (*P* > 0.05)***
	*PIK3C3-PTEN *	0.40 (*P* > 0.05)**	−0.25 (*P* > 0.05)***
	*PIM3-PTEN *	0.82 (*P* < 0.05)*	0.56 (*P* > 0.05)**

Liver	*PIK3C3-PIM3 *	0.49 (*P* > 0.05)**	0.54 (*P* > 0.05)**
	*PIK3C3-PTEN *	0.51 (*P* > 0.05)**	0.51 (*P* > 0.05)**
	*PIM3-PTEN *	0.82 (*P* < 0.05)*	0.79 (*P* < 0.05)*

Lung	*PIK3C3-PIM3 *	0.41 (*P* > 0.05)**	0.52 (*P* > 0.05)**
	*PIK3C3-PTEN *	0.44 (*P* > 0.05)**	0.38 (*P* > 0.05)**
	*PIM3-PTEN *	0.21 (*P* > 0.05)***	0.50 (*P* > 0.05)**

Ovary	*PIK3C3-PIM3 *	0.65 (*P* < 0.05)**	0.61 (*P* < 0.05)**
	*PIK3C3-PTEN *	0.71 (*P* < 0.05)*	0.77 (*P* < 0.05)*
	*PIM3-PTEN *	0.50 (*P* > 0.05)**	0.60 (*P* = 0.05)**

Prostate	*PIK3C3-PIM3 *		0.29 (*P* > 0.05)***
	*PIK3C3-PTEN *		−0.23 (*P* > 0.05)***
	*PIM3-PTEN *		0.56 (*P* > 0.05)**

Thyroid	*PIK3C3-PIM3 *	0.47 (*P* > 0.05)**	0.29 (*P* > 0.05)***
	*PIK3C3-PTEN *	0.11 (*P* > 0.05)***	−0.23 (*P* > 0.05)***
	*PIM3-PTEN *	0.75 (*P* < 0.05)*	0.58 (*P* > 0.05)**
