# Supplementary material for: Gene Expression Correlation for Cancer Diagnosis: A Pilot Study
Source: Biomed Res Int. 2014 Apr 9;2014:253804. doi: 10.1155/2014/253804 (PMC4000964; doi:10.1155/2014/253804)
Supplement: Supplementary file 1 — Patient demographic characteristics and cancer stage for the Origene TissueScan Oncology qPCR Cancer Survey Panel 96. [file 253804.f1.pdf]

Table S1: Patient demographic characteristics and cancer stage for the Origene TissueScan Oncology qPCR Cancer Survey Panel 96.

Patient Number	Tissue	Age	Gender	Stage	Patient Number	Tissue	Age	Gender	Stage
A01	Breast	44	F	0	E01	Lung	49	F	0
A02	Breast	45	F	0	E02	Lung	79	M	0
A03	Breast	32	F	0	E03	Lung	62	F	0
A04	Breast	47	F	I	E04	Lung	71	M	Ia
A05	Breast	52	F	I	E05	Lung	64	M	Ib
A06	Breast	57	F	IIa	E06	Lung	85	F	Ib
A07	Breast	42	F	IIa	E07	Lung	72	M	Ib
A08	Breast	63	F	IIIa	E08	Lung	63	M	IIb
A09	Breast	61	F	IIIa	E09	Lung	71	M	IIb
A10	Breast	55	F	IIIc	E10	Lung	58	M	IIIa
A11	Breast	45	F	IV	E11	Lung	80	M	IIIb
A12	Breast	42	F	IV	E12	Lung	51	M	IV
B01	Colon	61	M	0	F01	Ovary	70	F	0
B02	Colon	91	M	0	F02	Ovary	31	F	0
B03	Colon	37	F	0	F03	Ovary	42	F	0
B04	Colon	93	F	I	F04	Ovary	29	F	Ia
B05	Colon	65	M	IIa	F05	Ovary	43	F	Ib
B06	Colon	78	M	IIa	F06	Ovary	51	F	Ic
B07	Colon	66	M	IIa	F07	Ovary	80	F	IIb
B08	Colon	86	M	III	F08	Ovary	46	F	IIIa
B09	Colon	42	F	IIIb	F09	Ovary	52	F	IIIb
B10	Colon	61	M	IIIc	F10	Ovary	74	F	IIIc
B11	Colon	63	M	IIIc	F11	Ovary	77	F	IIIc
B12	Colon	51	F	IV	F12	Ovary	79	F	IV

C01	Kidney	71	M	0	G01	Prostate	68	M	0
C02	Kidney	66	M	0	G02	Prostate	65	M	0
C03	Kidney	54	F	0	G03	Prostate	76	M	0
C04	Kidney	52	M	I	G04	Prostate	70	M	I
C05	Kidney	55	F	I	G05	Prostate	63	M	II
C06	Kidney	52	M	I	G06	Prostate	70	M	II
C07	Kidney	57	F	II	G07	Prostate	71	M	II
C08	Kidney	59	M	III	G08	Prostate	56	M	II
C09	Kidney	37	M	III	G09	Prostate	63	M	II
C10	Kidney	64	M	III	G10	Prostate	53	M	III
C11	Kidney	70	M	IV	G11	Prostate	65	M	III
C12	Kidney	51	M	IV	G12	Prostate	61	M	III
D01	Liver	81	M	0	H01	Thyroid	30	F	0
D02	Liver	86	M	0	H02	Thyroid	68	F	0
D03	Liver	33	F	0	H03	Thyroid	46	F	0
D04	Liver	79	M	I	H04	Thyroid	15	F	I
D05	Liver	58	F	I	H05	Thyroid	28	F	I
D06	Liver	66	M	I	H06	Thyroid	39	F	I
D07	Liver	63	F	II	H07	Thyroid	57	M	II
D08	Liver	68	M	II	H08	Thyroid	74	M	II
D09	Liver	62	F	II	H09	Thyroid	76	F	III
D10	Liver	71	M	IIIa	H10	Thyroid	52	F	III

D11	Liver	21	M	IV	H11	Thyroid	52	M	IVa
D12	Bile duct	66	M	IV	H12	Thyroid	45	F	IVa
